# Applying a Support Vector Machine (SVM-RFE) Learning Approach to Investigate Students’ Scientific Literacy Development: Evidence from Asia, Europe, and South America

**DOI:** 10.3390/jintelligence12110111

**Published:** 2024-11-05

**Authors:** Jian Li, Jianing Wang, Eryong Xue

**Affiliations:** 1 Institute of International and Comparative Education, Beijing Normal University, Beijing 100875, China; 11112018031@bnu.edu.cn; 2 Research Institute of Science Education, Faculty of Education, Beijing Normal University, Beijing 100875, China; 3 China Institute of Education Policy, Faculty of Education, Beijing Normal University, Beijing 100875, China

**Keywords:** scientific literacy, science education, machine learning, support vector machine

## Abstract

Cultivating scientific literacy is a goal widely shared by educators and students around the world. Many studies have sought to enhance students’ proficiency in scientific literacy through various approaches. However, there is a need to explore the attributes associated with advanced levels of scientific literacy, especially the influence of contextual factors. In this context, our study employs a machine learning technique—the SVM-RFE algorithm—to identify the critical characteristics of students with strong scientific literacy in Asia, Europe, and South America. Our research has pinpointed 30 key factors from a broader set of 162 contextual factors that are indicative of outstanding scientific literacy among 15-year-old secondary school students. By utilizing student samples from the three continents, our study provides a comprehensive analysis of these factors across the entire dataset, along with a comparative examination of the optimal set of key factors between continents. The findings highlight the importance of these key factors, which should be considered by educational policymakers and school leaders when developing educational policies and instructional strategies to foster the most effective development of scientific literacy.

## 1. Introduction

Science education is vital in our increasingly scientifically and technologically advanced world, and students need to be scientifically literate and competent to fully participate and succeed in this society. In recent decades, considerable attention has been paid to the evaluation of students’ scientific literacy. The Programme for International Student Assessment (PISA), developed by the Organization for Economic Co-operation and Development (OECD), is an influential large-scale test designed to measure students’ ability to apply specialist knowledge and skills in practical scenarios. Students with strong scientific literacy excel at creating and applying knowledge. Therefore, investing in and educating robust scientific and technological innovators has become a crucial policy objective. Scientific literacy may demonstrate students’ innate talents but can also be related to contextual factors.

Several empirical studies using large-scale data analysis technology have been effective in elucidating the development mechanism and factors influencing middle school students’ literacy in specific subjects. For example, Chen and Xiao (2024) built a hierarchical linear model and used opportunity–propensity framework analysis to explore the effects of metacognitive reading strategies, reading self-concept information, and other factors on teenagers’ digital reading performance. Based on TIMSS data, Wiberg and Rolfsman (2021) explored the association between Swedish science test results and students’ school achievement. Studies have confirmed the contribution of specific factors to student performance based on the PISA dataset and drawing on several statistical analysis methods. A two-level regression conducted with data from PISA 2015 was used to analyze the differential relationship between students’ science performance and their ICT (information and communications technology) availability, ICT use, and attitudes towards ICT in South American, European, and Asian Pacific regions (Arpacı et al. 2021). An international comparative study analyzed PISA 2018 data from Turkey and China using the Automatic Rate Fallback (ARF) algorithm and found that students’ metacognitive ability and socioeconomic and cultural status are statistically significant factors for the academic performance of 15-year-old students in both countries (Kılıç Depren and Depren 2022). 

Machine learning involves instructing computers to run without explicit programming to intelligently acquire knowledge (Fahd et al. 2022). Applications for education have gradually been developed, now including student modeling, scientific inquiry, adaptive systems, assessment, and decision support systems (Bakhshinategh et al. 2018). Machine learning algorithms offer several distinct advantages over traditional data analysis methods when it comes to analyzing educational big data. Traditional research methodologies often rely on the assumption of random data generation to construct analytical models. These methods necessitate the prior establishment of a clear and comprehensive statistical framework, followed by the computation of specific model coefficients to illustrate the interrelationships among factors. In contrast, machine learning approaches acknowledge the complexity and unpredictability of data generation processes. They focus on observing the relationships between independent and dependent factors without presupposing a specific model structure (Senaviratna and Cooray 2019). Moreover, machine learning algorithms are adept at accounting for the interactions between the factors of interest and other potential confounding factors. Furthermore, machine learning excels at sifting through vast databases and numerous independent factors to identify the most significant predictors of a dependent variable. It presents these critical influencing factors to decision-makers in a manner that is both intuitive and actionable, thereby enhancing the decision-making process with data-driven insights. Much of the applied research on data analysis in the field of education has been facilitated by machine learning in recent years, and common algorithms include Random Forest, Decision Tree, XGBoost, Support Vector Machine, Neural Network, etc. (Acıslı-Celik and Yesilkanat 2023; Doz et al. 2023; Özbey and Kayri 2023). 

## 2. Research Questions

In this study, one machine learning method—support vector machine (SVM-RFE)—was used to identify several contextual factors from 162 factors that could be used to classify and predict the optimal key factors of high and low performers in a sample of 15-year-old middle school students. At the same time, the set of key factors was explored, and the influence of these factors on scientific literacy at the non-cognitive level was investigated. Therefore, this study had two research questions:

**Q1.** From the overall data selected, what are the influencing factors at the five levels of SVM that can screen out students with excellent scientific literacy?
**Q2.** What are the differences and similarities between the factors predicting students’ scientific literacy in PISA 2022 surveys in Asia, Europe, and South America as a whole and in different regions?

## 3. Analytical Framework

Walberg’s (1981) theory of educational productivity is a comprehensive theoretical framework that aims to explore factors affecting student learning and analyze how these factors affect student academic performance. This theory emphasizes the important influence of internal and external factors of the education system on student learning, arguing that it is impossible for educators to cultivate achievement through their own efforts without involving social contextual factors because learning is “an individual emotional, behavioral, and cognitive activity that mainly takes place in social contexts” (Walberg 1984). By analyzing more than 3000 studies, Walberg classified the factors affecting students’ academic performance into eleven areas, eight of which were affected by social–emotional factors, including classroom management, parental support, teacher–student interaction, social behavior factors, motivational factors, and peer groups. He emphasized that these factors, if properly managed and valued, can significantly affect students’ scientific literacy and academic performance (Walberg 1983). For example, good classroom management can provide students with an orderly learning environment, parental support can enhance student self-confidence and learning motivation, and positive teacher–student interaction can enhance students’ learning interest and engagement. 

With the long-term goal of balancing continuity and innovation, the PISA 2022 Context Questionnaire Framework retains the key framework elements of the previous PISA test cycle as a foundation. Improvements have been introduced to facilitate the development of new productivity (OECD 2023a). The PISA 2022 Context Questionnaire Framework covers individual-level factors, the teaching and learning environment in the educational process, and the broader education system and policy levels. The multi-dimensional analytical framework clearly reflects the ideological core of Walberg’s theory of educational productivity and aids in the comprehensive understanding of how factors interact in the process of education to jointly affect educational outcomes.

**Figure 1:**
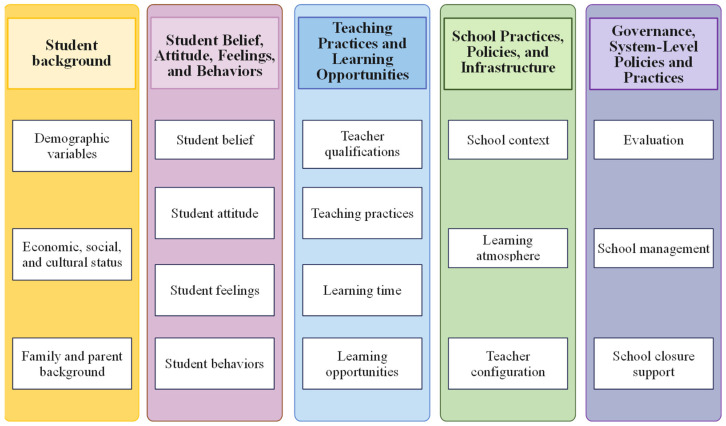
Classification of contextual factors into five domains with sub-domains.

Based on the above discussion, we extracted independent factors from student and school questionnaires of PISA 2022 to analyze factors that may affect students’ scientific literacy and academic performance. We classified the extracted factors into five dimensions to determine the key background factors that best distinguish between high and low scientific literacy (Figure 1). Some of these factors were extracted directly from the original questions, while others were developed from a combination of other questions using PISA 2022 data. 

### 3.1. Student Background

Individual identity factors, such as basic demographic variables; structures related to economic, social, and cultural status (ESCS); and family background (such as parents’ education level) must be considered to understand the context-level factors that affect students’ scientific literacy, educational pathways, and equity within and across educational systems. Among student-level contextual factors, a student’s socioeconomic status (SES) is often cited as having a profound effect on their academic performance. SES is typically expressed by investing in household resources. For example, a study in Hong Kong showed that parental investment in cultural resources and organizing early science enrichment activities were significantly correlated with students’ scientific literacy performance (Ho 2010). Topçu et al. (2014) noted that educational resources (e.g., electronic devices, textbooks), cultural capital (e.g., literature, poetry), and economic wealth (e.g., cars) were all positively correlated with student performance in their study comparing the scientific performance of Turkish students in PISA 2006 and 2009. However, it has also been argued that differences in academic achievement between students are not materially affected by SES factors. 

### 3.2. Students’ Beliefs, Attitudes, Feelings, and Behaviors

Measuring students’ subjective attitudes and feelings and their behavioral choices can provide important indicators of an education system’s success in producing productive members of society. Beliefs include students’ mindsets or beliefs about learning. Attitudes include a student’s attitude towards a subject area. Emotions relate to feelings about school or specific subject areas, as well as social and emotional factors. Behavior includes participation in out-of-school activities or behavioral aspects of social and emotional traits. Factors such as respecting and understanding others, being motivated to learn and cooperate, or being able to regulate one’s own behavior may be prerequisites for acquiring knowledge and skills in subject areas. PISA 2022 addresses a range of relevant factors, such as student effort on PISA tests and questionnaires, general school attitudes and feelings in relation to the school climate, attitudes towards specific content areas, general social and emotional factors, out-of-school experiences, and subjective perceptions of students’ SES and future aspirations and well-being. She et al. (2019) showed that belief in science knowledge, learning time, interest in a wide range of science topics, achievement motivation, an inquiring science teaching practice, and science self-efficacy can significantly predict students’ scientific literacy performance. At the same time, there is evidence that school bullying adversely affects the educational production process (Ponzo 2013)—being victimized or learning in a bullying atmosphere are significantly negatively correlated with student achievement in science, math, and reading (Huang 2020).

### 3.3. Teaching Practices and Learning Opportunities

Classroom teaching is the direct and core environment for formal and systematic education. Therefore, policymakers need information about the organization of the classroom and the teaching experience within it. Educational effectiveness research explores core factors that affect student achievement (Creemers and Kyriakides 2007), such as teaching practice, learning time, and learning opportunities provided in and out of school. An international comparative study based on data from six countries showed significant relationships between scientific literacy and instructional adaptation, perceived feedback, teacher inequality, teacher-directed science teaching, and inquiry-based science teaching and learning practices in most countries (Kalkan et al. 2020). The concept of “opportunity to learn” (OTL) indicates whether students have enough time and receive adequate learning instruction (Abedi et al. 2006). OTL may include subject-specific teacher guidance (Callahan 2005), teaching quality (Minor et al. 2015), in-school learning opportunities, and off-campus informal and formal learning opportunities. A review of 28 studies showed that time spent and the number of teaching days in the school year are broadly associated with student academic achievement, including in science subjects, although there may be a ceiling effect for teaching time (Yeşil Dağlı 2018). Among the PISA factors, OTL data include opportunities to learn through the way students are organized to learn, opportunities based on the learning content to which students are exposed, and opportunities based on the behavior displayed by teachers in the classroom.

### 3.4. School Practices, Policies, and Infrastructure

School-level factors (such as practices, policies, and infrastructure) contribute to improving schools, thereby indirectly improving student learning. This dimension mainly includes the environmental factors of a school, that is, the physical structural factors (such as SES, location, type, and resources of the school); factors of the school climate, i.e., the learning environment (e.g., disciplinary climate); and teacher allocation (e.g., teacher qualifications, number of teachers, and teacher–student ratio). Factors such as the collective effectiveness of teachers, principals’ leadership, and the school’s support of teaching are critical to creating a positive school climate conducive to learning. In terms of environmental factors, researchers have explored the influence of factors such as teaching practice management mode, scientific resource supply, and school SES on student learning (Tan et al. 2020). A disciplined atmosphere (Krskova and Baumann 2017), the provision of school learning resources, and space are factors of an atmosphere conducive to improving academic performance. As far as teacher allocation is concerned, some research indicate that reducing the teacher–student ratio can improve the quality of education, but there are also views that the reduction of class size has little or no effect on academic performance (Hoxby 2000). 

### 3.5. Government and School-Level Policies and Practices

To address policy requirements directly, PISA must also address issues related to system-level governance. Among them, assessment and evaluation are the basic processes used by decision makers and/or school administrators to control school quality and monitor and promote school improvement (Staman et al. 2014). The school’s decision-making responsibility, emphasis on assessment testing, and supervision of parental involvement in students’ education may also affect students’ learning outcomes and academic achievement. The global spread of COVID-19 caused unprecedented academic disruptions worldwide, and the forced closure of schools made it necessary for schools and education systems to create alternative learning opportunities for students to mitigate lost time and support, particularly to avoid increasing inequality in schools in “educationally poor” areas. Nigerian educators, for example, used radio as a medium to support young learners to continue their education in science and other subjects at a distance (Ebubedike et al. 2022).

## 4. Materials and Methods

Data mining methods are often used for data analysis when detecting and interpreting large databases with complex relationships among many factors. The two main types of data mining methods are supervised and unsupervised. When the topic is clear, a supervised approach is used to train a classifier that can effectively and efficiently generalize the topic classification to new datasets. In contrast, when the topic is unclear, an unsupervised approach is used to divide the topic into separate categories. We have tested several models, including SVM, Random Forest, Decision Trees, etc. The results of our analysis indicate that the factors provided by these models are similar, and in terms of evaluation metrics, the performance of SVM-RFE is the best. Considering the possible predictors in this study, we chose a supervised learning method (kernelized SVM, SVM-RFE). The SVM and the SVM-RFE methods have been successfully applied to the analysis of PISA datasets in several studies (Chen et al. 2019; Zheng et al. 2024).

### 4.1. Support Vector Machine (SVM)

SVM is a machine learning method based on statistical learning theory (Cortés and Vapnik 1995). SVM can be extended to more complex models that can be used for the classification of discrete dependent factors and the prediction of continuous dependent factors. The core mechanism of the model is to construct the “hyperplane” with the greatest margin using support vectors, that is, the segmentation plane. The learning strategy is to maximize the interval, and finally select the best hyperplane from countless segmentation planes to effectively separate the two types of samples.

Suppose there are *n* independent and identically distributed observation samples: $\left(x_{1},y_{1}\right),\cdots \left(x_{k},y_{k}\right)\epsilon R^{d}\times \left\{\pm 1\right\}.$

Here, $\;x_{i}(i=1,2,\cdots n)$ represents the input vector with d factors. For a binary classification problem, the class labels are $\left\{+1,-1\right\}$. The training pattern $\left\{x_{1},x_{2},{\cdots x}_{n}\right\}\;$ is used to establish a decision function or discriminant function F(x), and new vectors will be classified based on the sign of the decision function:

$$F\left(x\right)>0\Rightarrow x\;\epsilon \;class\left(+\right),\;F\left(x\right)<0\Rightarrow x\;\epsilon \;class\left(-\right),\;F\left(x\right)=0,\;decision\;boundary.$$

In a d-dimensional space, the linear discriminant function is $F\left(x\right)=\omega \cdot x+b$. The classification surface equation is ω·x+b=0, while ω is the weight vector and b is the bias term. 

SVM derives from the idea of the optimal classification plane in the case of linear separability. By normalizing the above equation, all samples from both classes to the classification surface satisfy $\left|d(x)\right|\geq 1$. If the classification surface can correctly classify all samples, it should satisfy the constraint $y_{i}\left(\omega \cdot x_{i}+b\right)-1\geq 0\;i=1,\cdots ,n$. We call the training data that satisfy this equation the support vectors (SVs). An important definition is the classification margin—the distance between the classification line and the line closest to the classification line after classification, parallel to the classification line: margin=2ω, with larger margins considered better. The classification surface that satisfies the following conditions and minimizes $\frac{1}{2}{\left\|\omega \right\|}^{2}$ is called the optimal classification surface, which has a training error rate of 0, can correctly separate the two classes of samples, and maximizes the classification margin.

The requirement is to minimize min12ω2, which can be achieved using the Lagrange method to solve its dual problem, a unique solution to a quadratic function optimization problem with inequality constraints. The resulting optimal classification function is:

$$f\left(x\right)=sgn\left\{\left(\omega \cdot x\right)+b\right\}=sgn\left\{\sum_{i=1}^{n}a_{i}^{*}y_{i}\left(x_{i}\cdot x\right)+b^{*}\right\}.$$

$a_{i}$ is the Lagrange multiplier corresponding to each sample, and in the solution, $a_{i}^{*}\neq 0$ corresponds to the training samples that are SVs. Typically, only a few Lagrange multipliers are non-zero. $b^{*}$ is the classification threshold, which can be calculated from any SV or the median of any two SVs from the two classes.

Given that not all problems are linearly separable, some training samples cannot be completely separated by the linear optimal hyperplane. Therefore, a relaxation term $\xi_{i}\geq 0$ is added to the above conditions to soften the hard margin constraints and improve the accuracy of classification. The modified condition is $y_{i}\left[\left(\omega \cdot x_{i}+b\right)\;\right]-1+\xi_{i}\geq 0,\;i=1,\cdots ,n$; thus, the objective is to find $\left(\omega ,\xi \right)=\frac{1}{2}{\left\|\omega \right\|}^{2}+C\left[\sum_{i=1}^{n}\xi_{i}\right]$ minimized. Here, C is called the penalty parameter, a positive constant that controls the degree of punishment for misclassified samples. Under the constraint ${0\leq a}_{i}\leq C,i=1,\cdots ,n$, the dual problem can be solved.

The value of C affects the predictive accuracy of the classifier, but its optimal solution is not easy to determine. Therefore, in practical applications, V-fold cross-validation testing is often used to determine the value and ensure the accuracy of the algorithm (Wong and Yeh 2020). Typically, the entire training set is divided into V equally sized subsets, and one subset is used as the test set while the remaining V-1 subsets are used as the training set, in turn. Each trial will yield the corresponding accuracy (or error rate), and the average of the V results will be taken as an estimate of the predictive accuracy and algorithm accuracy of the classifier. 

In the optimal classification plane, appropriate kernel functions $K(x_{i},y_{j})$ can be used to achieve linear classification after a certain nonlinear transformation. At this time, the objective function changes, and the corresponding classification function becomes:

$$f\left(x\right)=sgn\left(\sum_{i=1}^{n}a_{i}y_{i}K\left(x_{i}\cdot x\right)+b^{*}\right)$$

### 4.2. Support Vector Classification (SVC)

SVMs are used for Support Vector Classification (SVC). Python provides the implementation function of linear separable SVM or approximately linear separable SVM, which can be realized by importing the sklearn module and calling the SVC class in the SVM submodule. Like other classifiers, SVC takes two arrays as the input: an array X size as the training sample, and an array y size as the class label (string or integer). After fitting, the classifier model can be used to predict the class of new sample values. The SVM decision function depends on the subsets of the training set (support vectors), some of whose factors can be found in the support vectors_, support_, and support attributes.

### 4.3. Feature Selection: SVM-RFE

In the field of machine learning, feature selection is a key step to improve model performance and interpretability. The ideal feature selection for pattern classification is to screen the most influential feature sets. A good feature set should have the features of small number, high reliability, discriminability, independence, and stability. Recursive Feature Elimination (RFE) is a greedy search algorithm for wrapper models using feature ordering techniques for feature selection. The output result of feature selection can be divided into a feature subset and a sorted list. RFE starts from the entire set and eliminates the least relevant features one by one under certain feature ordering standards to realize the feature ordering and output the sorted list. Unimportant or irrelevant features are always the first to be eliminated, and therefore are the lower part of the list. In contrast, the most important features are always eliminated at the end, and thus are placed first on the list. Finally, RFE defines several nested feature subsets according to the sorted list of features, and then uses the prediction accuracy of the classifier as the evaluation standard of the feature subsets to obtain the optimal feature subset. SVM-RFE (Recursive Feature Elimination based on Support Vector Machine) is a feature selection method based on SVM (Guyon et al. 2002). It optimizes feature subsets by recursively removing features that contribute least to classification and is suitable for several types of datasets, including linearly separable and nonlinearly separable data. The basic principle of the SVM-RFE algorithm is to use SVM as a classifier to evaluate the importance of features according to the trained SVM coefficients. In SVM, only SVs have a direct influence on classification decisions, and other features have relatively little influence. By analyzing the weights of these support vectors, we can identify features that contribute less to the model and remove them from the feature set. This iterative process is performed recursively with backward elimination of features until a predetermined number of features is reached or other stopping conditions are satisfied.

### 4.4. Model Evaluation Indicators of SVM-RFE

Given that SVM-RFE is a feature selection algorithm for a wrapper model, the quality of the selected feature subset needs to be evaluated by the Accuracy, Precision, Recall, F1 score, AUC, and ROC of the SVM classification model. Accuracy is the overall percentage of correct prediction results, that is, the percentage of true-positive (TP) and true-negative (TN) data of prediction results in the test data, i.e., Accuracy = (TP + TN)/M, where M is the number of test set samples. Accuracy or accuracy rate is an evaluation index of classification models, mainly used to evaluate the prediction accuracy of models for positive cases. Recall rate, also known as sensitivity, is used to evaluate the model’s ability to cover positive cases. The F1 score is a weighted harmonic average of accuracy and recall providing a balanced assessment. The Receiver Operating Characteristic (ROC) curve comprises a horizontal axis that represents the False Positive Rate (FPR) and a vertical axis for the True Positive Rate (TPR). AUC (Area Under ROC Curve) represents the probability that the current classification algorithm will place the positive sample before the negative sample when the user randomly selects the two samples, which can be obtained by summing the area under the ROC curve. Under normal circumstances, 0.5 < AUC < 1.0, and values of AUC closer to 1 indicate that the SVM algorithm used to measure the “binary classification problem” has strong generalization ability and good performance.

### 4.5. Participants

The data source for this study was mainly the PISA 2022 database (https://www.oecd.org/pisa/data/2022database/, accessed on 30 November 2023). PISA is a global educational assessment program initiated and implemented by the OECD to assess the ability of 15-year-olds in reading, math, and science. Since its first implementation in 2000, PISA has become an important tool for measuring the quality of education worldwide. The PISA 2022 data derive from the most recently conducted assessment, covering a large volume of data from multiple countries and regions, processed through rigorous sampling and statistical methods to ensure its representativeness and scientific nature. We selected Asian, European, and South American datasets from the PISA 2022 database, including background information, academic performance, and relevant psychological and socioeconomic indicators of students. Through in-depth analysis of these data, this study aimed to explore the relationship between education quality and student ability development, providing a theoretical basis and empirical support for policy making and practice in related fields. Binary factors were used to represent student performance: top performers (PISA levels 5 and 6) and lower-level performers (below level 5). In PISA 2022, students with a plausible value (PV) of more than 633 were identified as top performers (OECD 2023b), comprising 8.7% of the sample. PISA 2022 uses 10 PVs to represent each student’s scientific literacy to provide an accurate estimate of their ability. According to the PISA Data Analysis Manual (OECD 2009), using one or five likelihood values does not make a material difference in a large sample of data. Therefore, the first PV (PV1SCIE) was randomly selected as each student’s science grade.

### 4.6. Analysis Procedure

First, the original data were preprocessed, and the factors at the student level and school level were integrated according to the student ID, that is, the factors in the corresponding school questionnaire were supplemented according to the factor “CNTSCHID” in the student questionnaire. Next, for the 356 factors in the student and school questionnaires, columns with more than 20% missing values in the sample (*N* = 20) and column factors that did not meet the scope of consideration in this study were deleted, and factors that needed to be merged were merged. All rows with missing values were deleted. Although it may cause partial data loss, this method maintains dataset consistency while controlling computational costs. The number of students available was counted and a CSV format dataset was generated. After preliminary processing of the data, 162 factors were used and a total of 42,315 student data were filtered. These students come from 4047 schools, including 1477 in Asia, 1736 in Europe, and 834 in South America. In view of the unbalanced proportion of student data between the two categories, student data were extracted from all available data by the random under-sampling method. One-third of the data were randomly chosen as the training set, and the remaining data formed the test set. This method was also applied for regional data processing. Both the training set and the test set were scaled so that the factor values of each column were respectively mapped to the same interval.

Next, the first 30 factors were input into the SVM model for pre-experiments to test whether SVM can distinguish between students with excellent and poor scientific literacy, adjusting the parameters that best meet the classification needs. Then, SVM-RFE was used to rank the significance of 162 contextual factors and output the optimal feature set in descending order. Finally, the performance of the model was evaluated according to the evaluation index of the model, and the data analysis results were visualized. 

In the formal research process, we first selected kernel functions and parameters for the SVM classifier model, and the performance of all SVM classification models in this study was evaluated with V-fold cross-validation to determine the optimal model. Some studies have shown that V = 10 can better estimate the error (Weiss and Indurkhya 1996). Here, the optimal penalty function C = 10 was calculated and determined with the optimal feature subset for the feature ordering list. 

To identify the classification significance degree of performers with low scientific literacy, SVM-RFE calculated the weight of each feature vector, sorted the features (key factors) according to the correlation between the feature vector and students with high scientific literacy, and eliminated the features with the least influence in each iteration, finally generating a descending list of several features. Therefore, SVM-RFE was applied to rank the importance of all 162 factors in the preprocessed PISA 2022 data for the degree of influence on the level of scientific literacy. In PISA studies, 20 to 30 features tend to comprise the optimal set (Gorostiaga and Rojo-Álvarez 2016). Therefore, the 30 key factors identified as features in this study were chosen as the best selection for this study. Given that the purpose of feature selection is to reduce the dimension of a feature set without reducing the classification accuracy, the minimum feature subset corresponding to the highest prediction accuracy is the optimal feature subset of the feature ranking list. Therefore, the SVM classifier was trained with L nested feature subsets ($F_{1}\subset F_{2}\subset {\cdots \subset F}_{L}$), and the prediction accuracy of each feature subset was evaluated with a SVM to obtain a prediction accuracy list corresponding to the sorted list and the nested subset. The value of L was the number of original data features—in this study, *L* = 162. The largest subset was the original feature set ${(F}_{L})$, and the smallest subset ${(F}_{1})$ contained only the last feature to be eliminated. 

In this study, all data preprocessing and analysis were performed with Python 3.5’s free public programs and a library of coding documents imported from scikit learn.

## 5. Results

### 5.1. Predictive Performance of the Model

**Figure 2:**
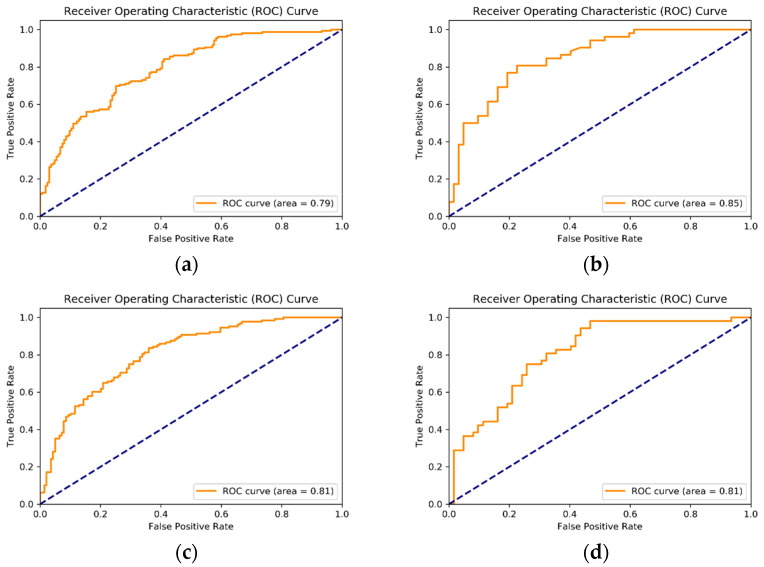
Receiver Operating Characteristic (ROC) curve through SVM-RFE. (**a**) The entire dataset (three continents); (**b**) Asia; (**c**) Europe, and (**d**) South America.

After training and parameter debugging, the SVM-RFE classification model finally adopted in this study performed well. The model was trained using the overall dataset and Asian, European, and South American datasets. The results of relevant evaluation indexes such as Accuracy, Precision, Recall, F1 score, AUC, and ROC of the SVM-RFE classification model are shown in Table 1 and Figure 2. The accuracy of all dataset test models was above 70.2%.

### 5.2. Key Factors for Scientific Literacy

#### 5.2.1. Key Factors of the Entire Dataset (Three Continents) 

Of the 162 contextual factors, there are 30 key factors that can distinguish between high school students who are good at science and those who perform less well, as shown in Table 2. 

Next, we compared the 30 contextual factors in Asia, Europe, and South America that best predicted and distinguished students with strong scientific literacy from those with a weaker ability. Using the country (CNT) factor, the student datasets were divided into three categories according to the continents to which the country belongs, and SVM classification was conducted for the datasets in different regions. The results were as follows.

#### 5.2.2. Common Key Factors of Asian and European Datasets

For key factor sets trained with Asian and European data, there were four overlapping key factors (Table 3).

#### 5.2.3. Common Key Factors of Asian and South American Datasets

For the key factor set trained with data from Asia and South America, there were eight overlapping key factors (Table 4).

#### 5.2.4. Common Factors of European and South American Datasets

For the key factor set trained with European and South American data, there were 10 overlapping key factors (Table 5).

## 6. Discussion

An analysis of PISA 2022 data revealed 30 key factors that together shape students’ superior performance in scientific literacy. These factors cover student background, family environment, learning attitude, feelings and behavior, teaching interaction, learning opportunities, socio–emotional background, educational background, and school policies, management, and practice. In this section, we will present a comprehensive review of the data from three continents, along with a comparative analysis of the results between these continents. Given the significant impact of scientific literacy on educational outcomes, our research aims to provide valuable insights that can inform the development of targeted educational policies and interventions.

Review of the overall data analysis results for three continents: Asia, Europe, and South America.

### 6.1. Key Factors of Student Background

For the key factors at the level of student background, the research found that the family’s digital equipment ownership is significantly correlated with students’ scientific literacy. Specifically, having a personal smartphone and the number of digital devices (e.g., televisions and e-book readers) in the home provides students with a wealth of information resources and learning tools that may facilitate their exploration and understanding of scientific knowledge. Technological devices, such as computers connected to the Internet, provide a platform for students to access scientific information and conduct online learning. In particular, the PISA 2022 test was conducted during the COVID-19 epidemic, and the popularization and technical support of such digital devices was particularly important for students to study at home. The closure of schools during the pandemic and the implementation of distance learning meant that digital devices in the home were key for accessing educational resources and for students to maintain a continuity of learning. Therefore, the quantity and quality of digital devices in the home may have had a significant effect on learning outcomes during this period.

The ownership of religious books may reflect the beliefs and values of the family, which to some extent shape students’ attitudes and interest in science. Parents’ educational level is also an important indicator of the academic atmosphere of the home, and higher parental educational levels suggest a greater likelihood of the family investing and supporting education, thus providing a more favorable learning environment for students. Family SES is also a key factor affecting students’ scientific literacy. The number of cars in the home and living conditions, such as the number of rooms with bathrooms or showers, are indirect indicators of the economic level of the household. Better-off families can provide their children with more learning resources and opportunities, which may help increase student motivation and their ability to learn science. In addition, the presence of “artistic” equipment such as musical instruments in the home may indicate family support for art and culture, which seems to be corroborated by the inclusion of “art” in STEAM education, indirectly promoting students’ curiosity and creativity in science. The phenomenon of students repeating grades may indicate that they have encountered academic difficulties, which could affect their self-confidence and negatively affect their science learning. Thus, repeating grades has become a key factor of students’ background level to distinguish between superior and non-superior scientific literacy. Gender, as a sociocultural factor, may also affect students’ attitudes and performance in science subjects. 

### 6.2. Key Factors of Student Beliefs, Attitudes, Feelings, and Behaviors 

First, a correlation was found between the number of paid days students work outside of school and their performance in science. Students involved in paid work may face challenges with time management and study pressure, which could affect their science studies. The increase in working days may reduce the time students spend studying and reviewing scientific knowledge, that is, students may have fewer learning opportunities, thus affecting their learning effectiveness in the field of science. Second, students’ self-cognition of science subjects has a significant effect on their learning attitudes and behaviors. Students who think science is easy tend to show higher motivation and more positive learning behaviors, which may help them achieve better grades in the science field. Conversely, students who perceive science as difficult may experience learning frustration that reduces their interest and motivation to learn. Student attitudes toward math are also closely related to their ability in science. Mathematics is the foundation of science subjects, and enjoying and excelling at mathematics can enhance students’ ability to solve scientific problems. Thus, students who consider math their favorite subject are likely to show stronger potential and higher achievement in science. School bullying has a negative effect on students’ socio–emotional well-being and ability to study. Students who experience bullying may face emotional distress and social exclusion, affecting their mental health, motivation to learn, and, ultimately, their scientific literacy as a result. The frequency and purpose of students’ use of digital resources in their spare time is also noteworthy. Although digital resources provide students with a wealth of learning materials and entertainment content, overuse or improper use may distract students and affect their learning efficiency. In particular, the use of digital resources for leisure activities in schools may reduce their opportunities to engage in scientific practice.

### 6.3. Key Factors of Teaching Practices and Learning Opportunities

The time spent on homework in the language subjects used in the PISA test is closely related to scientific literacy. Language skills are fundamental to scientific understanding and help students to interpret scientific texts and express ideas. Students who devote more time to language subjects tend to have stronger reading comprehension and information processing skills, more positive attitudes toward learning, and better time management and cognitive load handling—factors that may collectively contribute to their academic performance in the sciences. Interestingly, several of the key factors that distinguish science excellence from average performance at this level are also relevant to math teachers and math teaching practices. The proportion of mathematics teachers in schools is an important indicator of the quality of science education. An increase in the number of math teachers can mean more teaching resources and personalized guidance to help students build a solid foundation in math, which is essential for science learning. The improvement of math ability can enhance students’ ability to solve science problems, thus improving their academic performance in science. The professional training level of mathematics teachers directly affects their teaching methods and results. Well-trained math teachers are more likely to employ innovative and effective teaching strategies that stimulate student interest in math and science and improve their problem-solving skills. This improved mathematical ability can translate into greater scientific inquiry and analysis skills. Ability grouping in mathematics teaching may have a polarizing effect on students’ academic performance in science. Ability grouping ensures that students receive appropriate instruction according to their level and helps to improve learning efficiency. However, it can also lead to differentiation of literacy among students, limiting their scientific exploration at higher levels. Therefore, ability grouping should be implemented carefully to ensure that all students can improve their scientific literacy.

Positive teacher expectations can motivate students to study science, while professional development of teachers can provide richer teaching resources and methods to improve students’ scientific literacy. The proportion of faculty with at least an ISCED level 7 master’s degree reflects the overall academic level of the faculty. Highly educated teachers usually possess more in-depth subject knowledge and more advanced teaching concepts and can more effectively guide students in the cultivation of scientific inquiry and critical thinking, which may play a role in improving scientific academic performance. 

### 6.4. Key Factors of School Practices, Policies, and Infrastructure

The material conditions and policy environment of a school have a direct effect on students’ learning experiences. A shortage of educational resources may include insufficient science textbooks, laboratory equipment, or digital resources. This shortfall may directly affect students’ opportunities to acquire and apply scientific knowledge. Outstanding students may be more able to learn effectively with limited resources and demonstrate greater adaptability and problem-solving skills. Resource shortages may force students and teachers to adopt creative approaches to teaching and learning, and excellent students may exhibit higher levels of innovation and self-directed learning in this environment. In addition, high-performing students may be more able to positively influence and adapt to the school climate, for example, by actively participating in class discussions, science clubs, or competitions to enhance their scientific literacy. Interaction and cooperation among students also affect the learning atmosphere. Outstanding students may be more inclined to collaborate with their peers to share knowledge and experiences, thereby improving science learning for the entire group.

### 6.5. Key Factors in Governance and System-Level Policies and Practices 

Standardized tests provide a uniform assessment standard capable of objectively measuring students’ scientific knowledge and skills. These tests are often designed to cover a wide range of scientific concepts and competencies, thereby helping to identify students’ strengths and weaknesses in the sciences. Excellent students are often able to demonstrate higher scientific understanding and problem-solving skills on these tests. The proportion of school administrators may affect the allocation of school resources and the efficiency of management. An efficient administrative team can better support the professional development of teachers, ensure the rational allocation of teaching resources, and create an environment conducive to learning. This may indirectly affect students’ learning outcomes, including the development of scientific literacy. Outstanding students may benefit from more efficient administrative support, resulting in better results in science studies.

### 6.6. Comparative Analysis of Data from Asia, Europe, and South America

In Asia and Europe, students who are curious are more likely to do well in math classes, and this positive attitude helps them better understand complex concepts. Self-driven desire is key to student success, and students who want to do well in math classes tend to be more engaged and willing to take on challenges. Teachers continue to teach until students understand, showing a high standard of education quality and the importance of student understanding. The proportion of all teachers who are fully certified reflects the professionalism and quality of the education system. In Asia and Europe, a high proportion of certified teachers may mean higher quality teaching, which contributes to students’ scientific competence. An analysis of student samples from Asia and South America shows that the Economic, Social, and Cultural Status Index (ESCS) reflects a student’s family background, which can affect their access to and quality of education. 

In Asia and South America, students with higher status are likely to have more learning resources and support. At the same time, having their own room in the home may provide students with a quiet and focused learning environment, leading to better science performance. The perception that math is easier than other subjects may indicate that students have a higher level of confidence and interest in math. Given the relationship between math and science, this positive attitude may prompt them to put more effort into the scientific field. Similar to the situation in Asia and Europe, a self-driven desire to excel in mathematics is key to scientific excellence in Asia and South America. The duration of enrollment at a school may also reflect a student’s stability and degree of adjustment to the school environment, which may affect their learning outcomes. A shortage of educational staff may affect the quality of teaching and the individual concerns of students, and the type of school may affect the educational resources and learning environment provided, playing a differentiating role between those who excel in science in Asia and South America. The property index and the number of bathrooms or showers in the home may reflect a family’s living standard and economic status, and the number of classic literary texts in the home may indicate the family’s emphasis on culture and education, which could directly or indirectly affect a student’s learning environment, interests, and mentality.

The prevalence of smartphones in the home, the number of computers connected to the Internet, and the number of e-readers may provide students with access, opportunities, and tools to access information and learning resources—a factor in scientific excellence in both Europe and South America. Homework hours on the test language are also an indicator of scientific excellence in Europe and South America. Like the situation in Asia, this self-driven desire to excel in math is key to scientific excellence in Europe and South America. At the pedagogical level, consistent teaching and ability grouping in math classes may help to provide personalized instruction based on the needs of students, a strategy for improving scientific literacy in Europe and South America.

## 7. Conclusions

Students’ scientific literacy is multi-factorial. Families, schools, and society should work together to create a supportive and diverse learning environment. In this study, we focused on identifying key factors that can distinguish students with high scientific literacy from others. We also discussed the results from each continent and explored the similarities between the data from Asia, Europe, and South America. Educational policy makers and school administrators need to consider these factors comprehensively when formulating educational policies and teaching strategies to achieve comprehensive improvement of students’ scientific literacy. 

The innovative aspects and contributions of this research are highlighted in several key areas. Firstly, we have adopted the SVM-RFE technique to dissect the data, leveraging Recursive Feature Elimination to pinpoint the most impactful factors on exceptional scientific literacy. This approach is a relatively novel application within the realm of educational research, particularly when applied to extensive cross-national datasets. It surpasses traditional statistical methods by managing a vast array of factors and zeroing in on the key factors, potentially offering a more profound level of understanding. Secondly, while recent large-scale data analyses have often relied on older datasets, our study taps into the freshest data from PISA 2022. This allows us to capture the pulse of current educational landscapes, find the most recent trends and challenges, and uncover the latest determinants and mechanisms at play. Moreover, previous studies have predominantly concentrated on isolated factors, such as ICT influences (Arpacı et al. 2021), rather than integrating them into a comprehensive framework. Our research, however, takes a broader view by examining a spectrum of non-cognitive background elements, including family dynamics, school resources, and pedagogical practices. We have constructed a multidimensional analytical framework that aligns with Walberg’s educational productivity theory, offering a holistic lens through which to discern the critical attributes of students who excel in scientific literacy. Additionally, our study spans across Asia, Europe, and South America, providing a cross-cultural comparative perspective that is unique in the field. This approach stands in contrast to studies that have typically centered on specific regions or nations (e.g., Doz et al. 2023). It allows for a richer understanding of the factors influencing scientific literacy within diverse educational ecosystems.

In terms of practical implications, the findings of this study not only contribute fresh perspectives on the key traits of students with advanced scientific literacy but also furnish actionable insights for educational practitioners and policymakers. Regarding the limitations, we are aware that a single model may not fully capture all the complexities and nuances in the data. In future research, we hope to explore and compare the differences in handling such data with more machine learning models. Also, we recommend that future research should further explore the mechanisms of action of these factors in different cultures and educational systems and consider how to integrate these factors more effectively to promote the development of scientific literacy. Research should also focus on balancing the relationship between standardized testing and the overall development of students and stimulating students’ scientific interest and inquiry ability through educational innovation. Through this study, we call on the educational community to optimize the factors that affect students’ scientific literacy to cultivate more future talents with an innovative mindset and high levels of scientific literacy. Such cultivation is essential for the individual development of students and will have far-reaching significance for the overall progress and sustainable development of society.

## Figures and Tables

**Table 1:** Predictive performance of the datasets through SVM-RFE.

Optimal Dataset (30 Factors)	Accuracy	Precision	Recall	F_1_	AUC
The entire dataset (from three continents)	0.708	0.724	0.660	0.691	0.792
Asia	0.728	0.657	0.846	0.739	0.850
Europe	0.715	0.681	0.766	0.721	0.806
South America	0.702	0.632	0.827	0.717	0.809

**Table 2:** Key factors of the entire dataset (three continents).

Category	Ranking	Feature	Description
Student Background	1	ST250Q04JA	Which of the following are in your [home]: Your own [cell phone] with Internet access (e.g., smartphone)
	2	ST254Q01JA	How many of the following [digital devices] are in your [home]: Televisions
	3	ST254Q05JA	How many of the following [digital devices] are in your [home]: E-book readers (e.g., [Kindle™], [Kobo], [Bookeen])
	4	ST256Q01JA	How many of these books at [home]: Religious books (e.g., [Bible], [Example 2])
	9	PAREDINT	Index highest parental education (international years of schooling scale)
	12	ST251Q01JA	How many of these items are there at your [home]: Cars, vans, or trucks
	19	ST256Q07JA	How many of these books at [home]: Books on art, music, or design
	20	ST251Q03JA	How many of these items are there at your [home]: Rooms with a bath or shower
	21	RATCMP2	Computers connected to the Internet
	22	REPEAT	Grade repetition
	23	ST254Q06JA	How many of the following [digital devices] are in your [home]: [Cell phones] with Internet access (i.e., smartphones)
	24	ST251Q06JA	How many of these items are there at your [home]: Musical instruments (e.g., guitar, piano, [country-specific example])
	27	ST254Q03JA	How many of the following [digital devices] are in your [home]: Laptop computers or notebooks
	30	ST004D01T	Student (standardized) gender
Student Beliefs, Attitudes, Feelings, and Behaviors	5	ST294Q04JA	How many days/week before school: Work for pay
	7	ST268Q06JA	Agree/disagree: [Science] is easy for me.
	8	BULLIED	Being bullied (WLE)
	18	ST268Q01JA	Agree/disagree: Mathematics is one of my favorite subjects.
	26	ST326Q06JA	This school year, how many hours/day use [digital resources] for: leisure on weekends
	28	ST326Q04JA	This school year, how many hours/day use [digital resources] for: leisure at school
Teaching Practices and Learning Opportunities	6	ST296Q02JA	How much time spent on homework in: [test language] homework
	10	PROPMATH	Proportion of mathematics teachers at school
	13	MTTRAIN	Mathematics teacher training (WLE)
	15	ABGMATH	Ability grouping for mathematics classes
	16	TEACHBEHA	Teacher-related factors affecting school climate (WLE)
	29	PROPAT7	Proportion of all teachers with at least ISCED level 7 Master qualification
School Practices, Policies, and Infrastructure	11	EDUSHORT	Shortage of educational material (WLE)
	14	STUBEHA	Student-related factors affecting school climate (WLE)
Governance, System-Level Policies and Practices	17	STDTEST	Use of standardized tests (WLE)
	25	PROADMIN	Proportion of school administrative personnel

**Table 3:** Common factors of Asian and European datasets.

Category	Feature	Description
Student Beliefs, Attitudes, Feelings, and Behaviors	CURIOAGR	Curiosity (agreement) (WLE)
	ST268Q07JA	Agree/disagree: I want to do well in my mathematics class
Teaching Practices and Learning Opportunities	ST270Q04JA	How often does the teacher continue teaching until the students understand
School Practices, Policies, and Infrastructure	PROATCE	Proportion of all teachers fully certified

**Table 4:** Common factors of Asian and South American datasets.

Category	Feature	Description
Student Background	ESCS	Index of economic, social, and cultural status
Student Beliefs, Attitudes, Feelings, and Behaviors	MATHEASE	Perception of mathematics as easier than other subjects
	ST268Q07JA	Agree/disagree: I want to do well in my mathematics class
	ST226Q01JA	How long have you been enrolled at this school
	ST250Q01JA	Which of the following are in your [home]: A room of your own
Teaching Practices and Learning Opportunities	ST270Q04JA	How often does the teacher continue teaching until the students understand
Governance, System-Level Policies and Practices	STAFFSHORT	Shortage of educational staff (WLE)
	SCHLTYPE	School type

**Table 5:** Common factors of European and South American datasets.

Category	Feature	Description
Student Background	ST251Q03JA	How many of these items are there at your [home]: Rooms with a bath or shower
	ST256Q02JA	How many of these books at [home]: Classical literature (e.g., [Shakespeare], [Example 2])
	ST254Q06JA	How many of the following [digital devices] are in your [home]: [Cell phones] with Internet access (i.e., smartphones)
	RATCMP2	Computers connected to the Internet
	ST254Q05JA	How many of the following [digital devices] are in your [home]: E-book readers (e.g., [Kindle™], [Kobo], [Bookeen])
	HOMEPOS	Home possessions (WLE)
Student Beliefs, Attitudes, Feelings, and Behaviors	ST296Q02JA	How much time spent on homework in: [Test language]
	ST268Q07JA	Agree/disagree I want to do well in my mathematics class
Teaching Practices and Learning Opportunities	ST270Q04JA	How often does the teacher continue teaching until the students understand
	ABGMATH	Ability grouping for mathematics classes

## Data Availability

The data is available at https://www.oecd.org/pisa/data/2022database/, accessed on 30 November 2023).
